# Toxin‐Blocking Textiles: Rapid, Benign, Roll‐to‐Roll Production of Robust MOF‐Fabric Composites for Organophosphate Separation and Hydrolysis

**DOI:** 10.1002/cssc.202201744

**Published:** 2022-11-18

**Authors:** Sarah E. Morgan, Morgan L. Willis, Golnaz Dianat, Gregory W. Peterson, John J. Mahle, Gregory N. Parsons

**Affiliations:** ^1^ Chemical and Biomolecular Engineering North Carolina State University 911 Partners Way Raleigh North Carolina 27695 United States; ^2^ U.S. Army Combat Capabilities Development Command Chemical Biological Center 8198 Blackhawk Road Aberdeen Proving Ground Maryland 21010 United States

**Keywords:** inorganic chemistry, metal–organic frameworks, organophosphates, roll-to-roll, supramolecular chemistry

## Abstract

Current approaches to create zirconium‐based metal–organic framework (MOF) fabric composites for catalysis, water purification, wound healing, gas sorption, and other applications often rely on toxic solvents, long reaction/post processing times, and batch methods hindering process scalability. Here, a novel mechanism was reported for rapid UiO‐66‐NH_2_ synthesis in common low‐boiling‐point solvents (water, ethanol, and acetic acid) and revealed acid–base chemistry promoting full linker dissolution and vapor‐based crystallization. The mechanism enabled scalable roll‐to‐roll production of mechanically resilient UiO‐66‐NH_2_ fabrics with superior chemical protective capability. Solvent choice and segregated spray delivery of organic linker and metal salt MOF precursor solutions allowed for rapid MOF nucleation on the fiber surface and decreased the energy and time needed for post‐processing, producing an activated composite in less than 165 min, far outpacing conventional MOF‐fabric synthesis approaches. The MOF‐fabric hydrolyzed and blocked permeation of the chemical warfare agent soman, outperforming the protection‐standard activated carbon cloth. This work presents both chemical insights into Zr‐MOF powder and fabric composite formation by a rapid, industrially relevant approach and demonstrates its practicality and affordability for high‐performing personal protective equipment.

## Graphical abstract

**MOFs against toxins**: A scalable, rapid metal–organic framework (MOF)‐fabric production method is developed using benign solvents. The MOF‐composites are tested for lethal organophosphate neutralization and permeation for application to chemical warfare agent protective equipment.

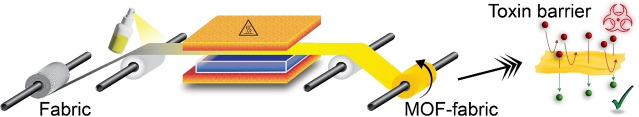

## Introduction

Polymer composites incorporating UiO‐type metal–organic framework (MOF) materials are proving useful for catalysis, environmental remediation, drug delivery, gas sorption, viral remediation, and other needs.[^1^, ^2^, ^3^, ^4^, ^5^] However, composite synthesis techniques reported to date are slow and environmentally unfriendly, relying, for example, on long batch reaction times, toxic solvents such as dimethyl formamide (DMF), and extended post‐processing steps, which hinder scalability and commercial implementation.[^1^, ^6^] As compiled in Table S1, researchers have explored nontoxic solvents including aqueous acetic acid or formic acid, but a key problem for UiO‐type MOFs is that common benzenedicarboxylic acid (BDC) linkers such as BDC, BDC‐NH_2_, and BDC−Br are generally insoluble in water, and introducing zirconium salts to form the metal secondary building unit (SBU) clusters can lead to rapid metal hydrolysis, undesired heterogeneous reactions, and viscous products.[^7^, ^8^, ^9^, ^10^, ^11^] In the case of BDC‐NH_2_, a disodium 2‐amino‐terephthalate precursor can be formed to increase aqueous solubility.^[12]^ Environmentally benign γ‐valerolactone (GVL), cyrene, ionic liquids, and other high‐boiling‐point solvents[^13^, ^14^] can help address this problem, but they also are not readily scalable because they add substantial cost and are difficult to remove from MOF pores after synthesis.[^14^, ^15^, ^16^, ^17^] Also, compared to MOF powders, producing polymer‐UiO‐66‐NH_2_ composites is more complex, often requiring long reaction times, lengthy post‐processing steps, and highly acidic environments.[^1^, ^9^, ^18^, ^19^, ^20^, ^21^] For example, we recently reported an environmentally benign sorption‐vapor method for MOF‐fabric composite synthesis using GVL as a green solvent, but samples were produced piece‐wise, where each 2”×2” MOF‐fabric piece underwent reaction for 24 h and post‐process cleaning for 72 h before undergoing testing.^[9]^ Table S2 lists previously reported in situ fiber‐first made UiO‐66‐NH_2_ MOF‐fabrics.

To enable more facile production of MOF‐composites, several researchers have explored roll‐to‐roll MOF‐fabric processing, but reported processes relied on toxic solvents, high processing temperatures, or were not applied to Zr‐type MOFs.[^22^, ^23^, ^24^] Therefore, there is a need to develop more versatile, scalable processing techniques for rapid, continuous, and environmentally safe roll‐to‐roll MOF‐fabric synthesis.[^1^, ^6^]

In this work, we report a novel aqueous‐based UiO‐66‐NH_2_ synthesis route for rapid, high‐yield production of MOF‐polymer composites. As an example system, we chose UiO‐66‐NH_2_‐composite polyester fabrics because these MOF‐fabrics have excellent performance for hydrolysis of toxic organophosphates including methyl paraoxon (DMNP) pesticides and chemical warfare agents (CWAs) including soman (GD), VX, and others.[^1^, ^9^, ^21^, ^25^, ^26^] This synthesis route is also amendable to other fabric types including spandex/polyester blends and polyamide. Our approach involves dissolving the linker and metal solutions in separate solvent mixtures and introducing them separately to the polymer composite substrate where they absorb into the polymer. This separated dissolution allows freedom in aqueous solvent choice and avoids undesired, premature homogeneous MOF nucleation. After reactants absorb into the polymer, the fabrics are exposed to water vapor at 120 °C allowing reactant out‐diffusion and rapid heterogeneous MOF crystallization, with the resulting MOF crystals robustly integrated onto the polymer surface. The use of water, ethanol, and acetic acid has a twofold benefit; they (1) promote rapid crystallization, and (2) volatize rapidly so that MOF pore activation times can be reduced to less than 2 h. Further, this method, using separated reactants, environmentally benign solvents, rapid heterogeneous nucleation, and minimal post‐reaction processing time, can be readily implemented for roll‐to‐roll MOF‐fabric synthesis, yielding MOF‐fabric materials with functional performance matching or exceeding those fabricated by more conventional, slower processes.

These UiO‐66‐NH_2_‐fabrics were evaluated for capture and solid‐state hydrolysis of chemical warfare nerve agents. For these fabrics, a single layer fully degraded soman in 24 h, and three layers achieved a soman breakthrough time of 400 min exceeding the standard activated carbon (AC) cloth. The chemical insights provided by this work expand Zr‐MOF fabric synthesis to industrially relevant roll‐to‐roll techniques therefore broadening the potential of MOF‐fabric applications. Cost evaluations indicate our materials are a significant step towards next‐generation, affordable personal protective equipment, which is important now more than ever due to heightened CWA threats.

## Results and Discussion

### Rapid, green UiO‐66‐NH_2_ synthesis mechanism

**Figure 1:**
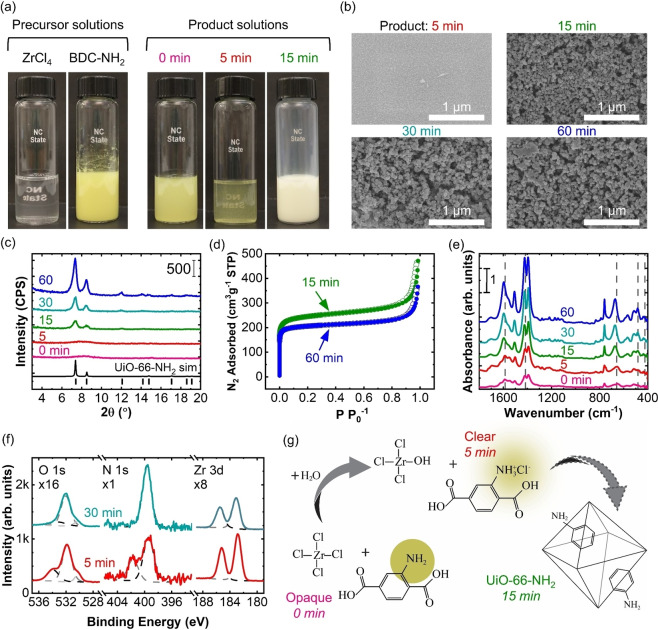
(a) Optical images of UiO‐66‐NH_2_ precursor and product solutions. (b) SEM images, (c) XRD, (d) N_2_ isotherms, (e) FTIR spectra, and (f) XPS spectra of UiO‐66‐NH_2_ powder products. (g) Proposed mechanism for BDC‐NH_2_ solvation by HCl.

Evolution of UiO‐66‐NH_2_ precursors into crystalline MOF in benign solvents was studied by characterizing the products formed when the metal salt (ZrCl_4_) reacted with the organic linker (BDC‐NH_2_) for various times. At room temperature, the ZrCl_4_ dissolves readily in ethanol (EtOH), producing a clear solution as shown in Figure 1a. At 90 °C, the BDC‐NH_2_ organic linker was sonicated in water/acetic acid (H_2_O/HOAc), producing a bright‐yellow opaque solution. Equal parts of the ZrCl_4_ and BDC‐NH_2_ solutions were mixed, heated to 90 °C, and the solution was observed as a function of time. Resulting images are given in Figure 1b. After different reaction times, 100 μL of the product solution was extracted and dispersed onto a silicon wafer and dried at room temperature for scanning electron microscopy (SEM), X‐ray diffraction (XRD), Fourier‐transform infrared (FTIR) spectroscopy, and X‐ray photoelectron spectroscopy (XPS) analysis. After 15, 30, or 60 min of reaction, the remaining solution was filtered, and the resulting powder product was collected, washed, and dried for N_2_ isotherm measurements.

As shown in Figure 1a, immediately after combining the linker and metal solutions at room temperature (0 min), the product solution was opaque and bright yellow. After 5 min at 90 °C, the product solution became clear with a yellow tint, and the SEM image in Figure 1b shows that the product was a dense solid with no defined crystals. After 15, 30, and 60 min at 90 °C, the product solution returned to an opaque appearance with a pale‐yellow precipitate. After 15 min, SEM shows defined crystals around 36 nm in diameter (measured by ImageJ). After 30 min, the crystals grew to around 57 nm and retained their size and shape after 60 min. Consistent with SEM images, XRD spectra show the 0 and 5 min products were mostly amorphous with a slight broad peak between 6–10° (Figure 1c). After 15 min, prominent peaks appeared at 7.3, 8.5, and 12.1° consistent with UiO‐66‐NH_2_ crystals, and the peaks became more intense after 30 min, consistent with larger crystal size. XRD intensity also increased from 30 to 60 min indicating rearrangement into a more ordered form while maintaining crystal size. The N_2_ isotherms of the precipitated products at 15, 30, and 60 min (Figure 1d) showed a Brunauer—Emmett—Teller (BET) surface area (SA) of 950, 1000, and 800 m^2^ g^−1^, respectively, further verifying the formation of porous UiO‐66‐NH_2_. The products after 15, 30, and 60 min also showed large N_2_ uptake at high relative pressure, consistent with microscale spacing between the nanoscale crystals.^[9]^

Figure 1e shows FTIR spectra of the products demonstrating how the chemical reaction evolved with time. The peaks at 424 and 480 cm^−1^ corresponding to μ_3_‐OH stretching increased in intensity up to 60 min, consistent with the formation of Zr clusters.^[20]^ The initial spectrum (0 min) shows the Zr−O stretching mode at 659 cm^−1^, and −OCO− asymmetric and symmetric stretching modes at 1417 and 1584 cm^−1^. After 60 min, the Zr−O peak shifted to 670 cm^−1^ and the −OCO− modes shifted to 1423 and 1596 cm^−1^, respectively. These shifts are consistent with −OH groups replacing the more electronegative Cl^−^ groups at the Zr node, and the expected −OCO− linker group deprotonation and incorporation into the MOF structure.[^27^, ^28^]

To further characterize chemical composition, we performed XPS on the 5 and 30 min products, and results are given in Figure 1f. Both samples show similar Zr 3d spectra with peaks associated with Zr−O clusters.[^29^, ^30^] The N 1 s peaks near 401.8 and 399.5 eV correspond to −NH_3_^+^ (protonated amine group) and −NH_2_, respectively,^[31]^ and peaks in the O 1 s spectra at 534.0, 531.8, and 530.3 eV correspond to −COOH, Zr−O−C, and Zr−O, respectively. Between 5 and 30 min, the protonated amine peak becomes substantially diminished and the Zr−O−C peak becomes more dominant, consistent with deprotonation of the BDC‐NH_3_^+^ linker and formation of UiO‐66‐NH_2_ precipitate.[^32^, ^33^]

From the data in Figure 1a–f, we hypothesize that the reaction between the MOF precursors follows the mechanism shown in Figure 1g. First, ZrCl_4_ hydrolyzes to form a Zr‐oxo complex releasing hydrochloric acid (HCl); the liberated HCl then protonates the BDC‐NH_2_ to form BDC‐NH_3_^+^Cl^−^ salt, which is soluble in the aqueous phase. Upon dissolution in the aqueous phase, the organic and metallic species react and stabilize as an insoluble UiO‐66‐NH_2_ crystalline precipitate. The zirconium cluster formation and carboxylic deprotonation is consistent with the observed shift in the O−C−O related peak in the FTIR data as well as the peak at around 401.8 eV in the XPS N 1 s peak associated with the protonated amine on the linker. As the reaction proceeds, the UiO‐66‐NH_2_ precipitate particles continue to grow and rearrange into a more crystalline product, as observed in SEM and XRD.

To test this proposed mechanism, we dissolved BDC and BDC‐NH_2_ in heated HCl solutions. Based on the mechanism, the BDC‐NH_2_ is expected to dissolve readily, whereas the BDC without the amine ligand will show limited dissolution. As shown in Figure S1a, adding BDC‐NH_2_ to a mixture of H_2_O/HOAc/EtOH/HCl at 90 °C led to a fully dissolved, clear solution, whereas the same concentration of BDC produced a poorly dissolved, opaque solution consistent with our hypothesis. This difference is understood by considering that in the aqueous HCl solution, the BDC‐NH_2_ readily forms an intermediate soluble BDC‐NH_3_^+^Cl^−^ salt, whereas the BDC (without the NH_2_ group) remains insoluble. Completing the UiO‐66 synthesis using BDC solution led to few MOF crystals and substantial unreacted precursor as shown by XRD in Figure S1b. These results indicate that for UiO‐66‐NH_2_, once the linker and metal solutions are mixed and heated, the linker dissolves into the aqueous phase through acid–base interaction. The facile reactant mixing in the aqueous phase then enables rapid reaction to form highly crystalline MOF.

### Benign, rapid MOF‐fabric production

#### Source‐separated sorption‐vapor synthesis of UiO‐66‐NH_2_‐fabric

Following the mechanism in Figure 1g, we hypothesized that UiO‐66‐NH_2_ MOF‐polymer composites could be formed rapidly by delivering the source reactants in separate solutions via spraying onto a polymer fabric substrate, followed by exposure to heated water vapor for heterogeneous MOF growth.[^9^, ^34^] We refer to this approach as “source‐separated sorption‐vapor synthesis” (SS‐SVS). Also, because the use of low boiling point solvents allows rapid precursor consumption to create nano‐scale MOF crystals, we further hypothesize that the solvent‐exchange step can be eliminated, thereby allowing much faster production of ready‐to‐use MOF‐fabrics.

**Figure 2:**
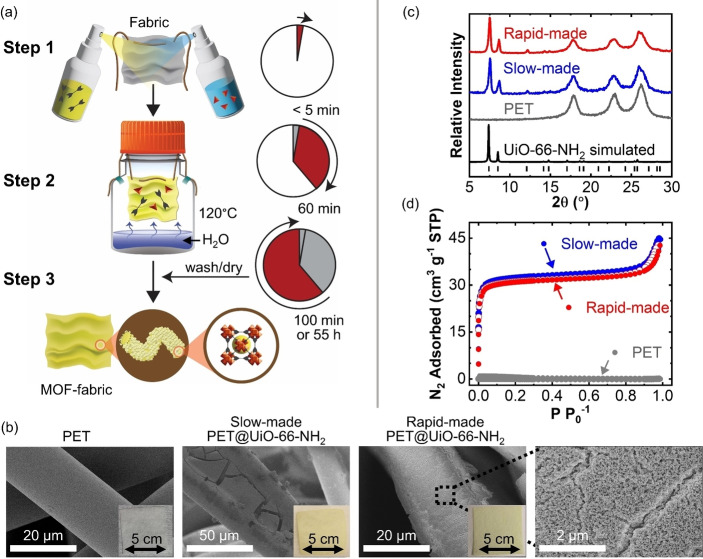
(a) Schematic of source‐separated sorption‐vapor synthesis of MOF‐fabric. (b) SEM images, (c) XRD, and (d) N_2_ isotherms of PET and slow‐ and rapid‐made PET@UiO‐66‐NH_2_.

To test the SS‐SVS method, metal EtOH/ZrCl_4_ (room temperature) and linker H_2_O/HOAc/BDC‐NH_2_ (90 °C) solutions were prepared and maintained in separate vials, as shown schematically in Figure 2a. Then (Step 1) a 5 cm ×5 cm (2 in ×2 in) polyethylene terephthalate (PET) fabric swatch was simultaneously sprayed with linker and metal solutions. This spraying step proceeded for around 2 min, during which around 2.6 mL of each solution was delivered approximately uniformly across the fabric sample. Fabric swatches were immediately moved to Step 2 without washing. Next, (Step 2) the fabric was suspended in a media vial over room temperature water, then transferred to an open‐air oven pre‐heated to 90 or 120 °C, allowing the vapor to interact with the soaked fabric for times ranging from 0.5–3 h. The solvent vapor assists precursor mobility and promotes MOF crystal formation.[^34^, ^35^, ^36^] Finally, (Step 3) the resulting PET@UiO‐66‐NH_2_ MOF‐fabric was allowed to cool for around 10 min at room temperature, then removed from the vial and washed and dried using a conventional procedure consisting of soaking in EtOH for 18–24 h, drying in air at 75 °C for 18–24 h, and drying under vacuum at 85 °C for 18–24 h. The total time for this post‐processing step (Step 3), referred to here as the “slow‐wash” method, ranged from 54–72 h.

For the different water vapor exposure temperatures and times in Step 2, the amount of MOF integrated onto the fabric was analyzed by mass, and the results are shown in Figure S2. The different exposure times and temperatures led to similar mass loading, whereas increasing vapor exposure time or temperature led to an increase in MOF crystallinity, as indicated by the XRD results in Figure S2c. This increased crystallinity is consistent with results from UiO‐66‐NH_2_ powder synthesis shown in Figure 1.

The resulting PET@UiO‐66‐NH_2_ MOF‐fabric was also tested for its ability to hydrolyze pesticide DMNP in an aqueous buffered solution. DMNP experiments were performed using *N*‐ethylmorpholine (NEM) as a basic co‐catalyst. Consistent with increased MOF crystallinity, fabrics produced using longer vapor exposure times and higher temperatures showed more rapid catalytic DMNP hydrolysis. For further SS‐SVS MOF‐fabric syntheses described below, we fixed the water vapor exposure conditions at 1 h and 120 °C, which produced materials with fast DMNP hydrolysis kinetics (*t*_1/2_≈23 min).

#### Comparing conventional and rapid post‐processing methods of MOF‐fabrics

In MOF‐fabric syntheses, the “slow‐wash” post‐process step described above is routinely used to ensure: (1) the commonly used high‐boiling‐point solvents such as DMF are sufficiently exchanged with low‐boiling‐point solvents such as EtOH; (2) any unreacted precursor and loose MOF powder are removed; and (3) the functional sites within the MOF crystal are fully activated for the desired application. Even with extensive washing and drying, high‐boiling‐point solvents such as DMF and GVL are known to remain in the MOF pores.^[9]^

As mentioned above, we hypothesize the SS‐SVS method can eliminate the need for solvent exchange, thereby reducing the time and energy required to activate the MOF. To test this hypothesis, we developed a new “fast‐wash” post‐process step for MOF‐fabric composites and compared the resulting composites to those produced with the “slow‐wash” procedure. The fast‐wash process consisted of two 5 min EtOH soaking steps and a 60 min drying step at 75 °C, followed by a 30 min drying step at 85 °C under vacuum. For the 5 cm ×5 cm sample used here, the fast‐wash method allowed MOF‐fabric to be fabricated to completion (i. e., ready for immediate use) in less than 165 min, which is 20× faster than the around 55 h needed to produce a similar MOF‐fabric material following the conventional, slow‐made process, and nearly 10× faster than previously reported in situ fiber‐first UiO‐66‐NH_2_ fabrics (Table S2).^[1]^

The slow‐made and rapid‐made MOF‐fabrics were analyzed by mechanical robustness testing, weight change, SEM, XRD, N_2_ adsorption, and organophosphate pesticide DMNP hydrolysis, and results are given in Figure 2b–d and Table 1. In both the slow‐wash and fast‐wash methods, the EtOH used to wash the MOF‐fabric after synthesis remained clear as shown in Figure S3 indicating minimal presence of unreacted precursor. The robustness of rapid‐made PET@UiO‐66‐NH_2_ was tested by laboratory handling, a tape test, and an abrasion testas shown in Video S1, with details given in the Experimental Section. As shown in the video, after common lab handling, no MOF material was visible on the nitrile gloves. Likewise, the tape test showed negligible MOF removal from the fabric. The abrasion test involved vigorously shaking the MOF‐fabric for 1 min in the presence of metal balls, after which the fabric sample showed a small amount of mass loss. Mass measurements indicated that 82 % of the original MOF remained on the fabric illustrating good mechanical resiliency. As shown in the SEM images in Figure 2b, following the rapid‐ PET@UiO‐66‐NH_2_ synthesis, the smooth starting PET fibers become covered with a dense, creviced crystal coating, and high‐magnification images reveal the coating is composed of nano‐scale crystals similar to the slow‐made materials.

**Table 1:** Select properties of slow‐made and rapid‐made PET@UiO‐66‐NH_2_. The uncertainty is the standard deviation for three MOF‐fabrics samples produced independently using identical conditions.

Synthesis	MOF loading [wt %]	BET SA [m^2^ g_comp_^−1^]	Porous MOF loading [wt %]	DMNP *t*_1/2_ [min]	DMNP TOF_*t*1/2_ [min^−1^]
slow‐made	16±1	123±14	15±2	23±12	0.3±0.1
rapid‐made	17±1	117±15	14±2	18±4	0.3±0.1

From the data in Figure 2 and Table 1, using the same starting materials, the MOF‐fabrics produced via the rapid‐made and slow‐made methods show similar MOF loading, N_2_ isotherms, BET SA, and XRD patterns. The rapid‐made and slow‐made fabrics also showed similar fast DMNP hydrolysis kinetics. The high performance of the rapid‐made composites confirms our hypothesis that MOF‐fabrics can be made using high‐throughput SS‐SVS batch synthesis while maintaining material properties and performance.

Spandex/polyester, cotton, polyamide, and polypropylene were also explored as substrates for rapid UiO‐66‐NH_2_ SS‐SVS, and the experimental conditions and visual, weight, and surface area results are presented in Figure S4. PET had the highest MOF loading and BET SA of the fabrics studied. We expect backbone chemistry and other factors such as fiber packing, fiber diameter, and fabric properties (i. e., woven vs. nonwoven) can impact the MOF crystallization during SS‐SVS. Further, based on previous sorption‐vapor synthesis work, we expect that MOF loading can be adjusted by either repeating SS‐SVS on the same fabric swatch or by adjusting the precursor concentration.[^9^, ^34^]

To test if the rapid‐made PET@UiO‐66‐NH_2_ fabrics can be re‐activated for DMNP hydrolysis, the same 14 mg MOF‐fabric swatch was used for two sequential DMNP hydrolysis experiments. As shown in Figure S5, the second experiment showed DMNP hydrolysis, but the rate was reduced compared to the first test. We also find that the rigorous stirring during the first 90 min hydrolysis step led to loss of MOF from the fabric, and the slower DMNP hydrolysis is consistent with the smaller MOF loading.

The XRD of PET@UiO‐66‐NH_2_ after DMNP hydrolysis was also collected. As shown in Figure S6, the UiO‐66‐NH_2_ crystallinity is maintained. The loss in peak intensity is consistent with the decrease in mass measured after the DMNP hydrolysis experiments.

#### Roll‐to‐roll MOF‐fabric production

**Figure 3:**
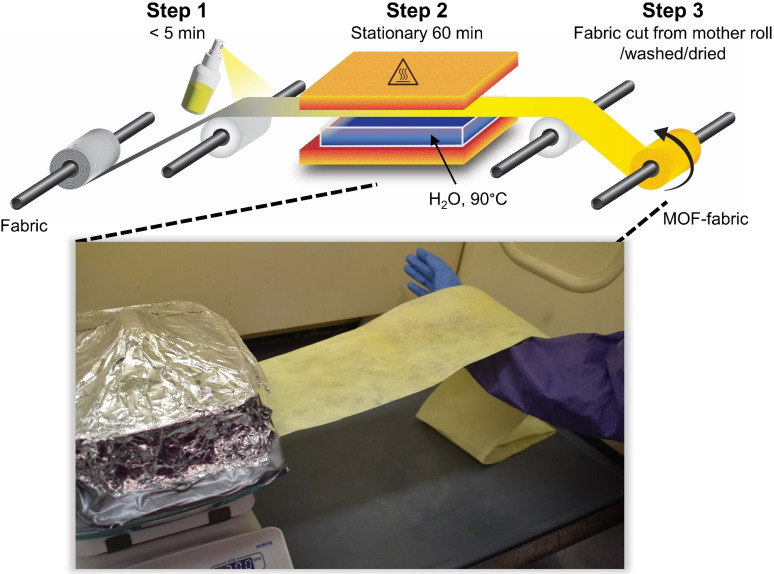
Roll‐to‐roll production system for MOF‐fabric and roll‐to‐roll made PET@UiO‐66‐NH_2_.

To demonstrate feasibility of process scaling, we evaluated the SS‐SVS method in a roll‐to‐roll configuration, as shown in Figure 3. The test configuration consists of a 15 cm wide nonwoven PET fabric rolled onto a feed spool, enabling fabric transport into an around 20 cm long spray zone. Within the spray zone, 40 mL precursor solution is applied, and the fabric is then immediately rolled into the heated crystallization zone (≈100 °C) where it is exposed to water vapor under static (stationary) conditions for 1 h. The MOF‐fabric is then rolled out of the crystallization zone and collected onto the final product roll. The sequence corresponded to a linear production rate of around 0.2 m h^−1^. For the fabric width of 15 cm used here, the linear rate corresponds to an areal rate of 0.03 m^2^ h^−1^. For analysis, a MOF‐fabric piece was cut from the fabric roll, washed in EtOH 10 min, and dried 24 h at 75 °C followed by 1 h at 85 °C under vacuum. Full details are given in the Experimental Section. The roll‐to‐roll made PET@UiO‐66‐NH_2_ (referred to as R2R PET@UiO‐66‐NH_2_) was analyzed for MOF adhesion and by SEM, N_2_ isotherm, XRD, and DMNP hydrolysis. Measurements taken in triplicate were from three 5 cm ×5 cm squares cut from the center of the roll, evenly distributed along the length.

The UiO‐66‐NH_2_ remained adhered to the R2R PET substrate during regular laboratory handling. A tape test revealed no MOF detachment from the fabric similar to the rapid‐made PET@UiO‐66‐NH_2_. Exfoliation could be forced by abrupt mechanical disturbances such as tearing. Generally, protective garments utilize multi‐layer fabrics, where the reactive layer (i. e., MOF‐fabric) is encased between two protective layers, thereby helping to avoid abrasion, tearing, sweat and other environmental concerns.^[37]^ SEM images, XRD, and N_2_ isotherm data (Figure S7a–c) confirm the formation of porous UiO‐66‐NH_2_ crystals on the PET surface and a composite BET SA of 250±75 m^2^ g_comp_^−1^. Roll‐to‐roll PET@UiO‐66‐NH_2_ hydrolyzed DMNP with a *t*_1/2_ of 13±5 min shown in Figure S4d, consistent with previous results. The MOF‐fabric made using the roll‐to‐roll system had a higher BET SA and slightly improved DMNP kinetics than the batch‐made PET@UiO‐66‐NH_2_, possibly due to the volume of precursor solution used: around 0.26 mL cm^−2^ roll‐to‐roll vs. around 0.20 mL cm^−2^ batch. These results represent pioneering work in scaled production of Zr‐MOF fabrics using roll‐to‐roll production. We calculated the materials cost needed to produce 15 cm wide PET@UiO‐66‐NH_2_ using our lab scale roll‐to‐roll set up. Without optimization, the estimated material cost of our MOF‐fabrics is $50 per linear meter (LM), which is less than the approximate price of activated carbon cloth by online suppliers of around $80 LM^−1^. This estimated cost was based only on the raw materials used to produce PET@UiO‐66‐NH_2_ and does not include indirect costs associated with manpower, facilities, and overhead.

### MOF‐fabric as a chemical warfare agent barrier: soman solid‐state hydrolysis and permeation

**Figure 4:**
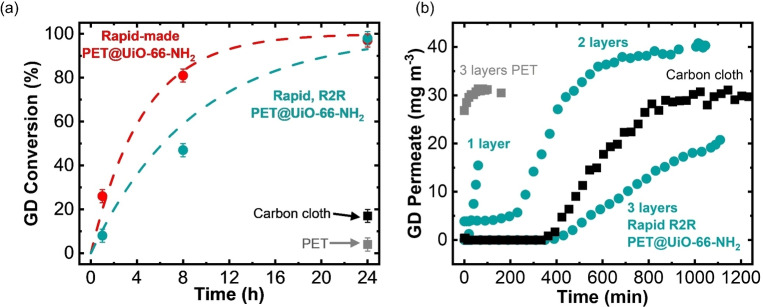
Soman (GD) (a) hydrolysis and (b) permeation tests with carbon cloth, PET, and PET@UiO‐66‐NH_2_. The dashed lines in panel (a) are exponential trend lines drawn as a guide to the eye. For the GD conversion data, error bars correspond to a predicted ±3 %, obtained when experiments are repeated on a duplicate sample.

Our batch rapid‐made and roll‐to‐roll made PET@UiO‐66‐NH_2_ fabrics were tested for soman (GD) hydrolysis and permeation and were compared to the starting fabric and the standard carbon cloth. The standard carbon cloth was provided by the U.S. Army Combat Capabilities Development Command Chemical Biological Center and has a density of 172 g m^−2^ and BET SA of 630 m^2^ g^−1^. Solid‐state hydrolysis experiments consisted of exposing live agent soman to the MOF‐fabric without any solution or buffer present. Conversion of GD as a function of time is shown in Figure 4a. Dashed lines in Figure 4a represent pseudo first‐order kinetics, and error bars represent a predicted ±3 % variation when the experiment is repeated with a duplicate sample. Rapid‐made and roll‐to‐roll made PET@UiO‐66‐NH_2_ fully degraded the GD in 24 h, outperforming untreated PET and carbon cloth, which showed limited reactivity in 24 h. Permeation of GD through roll‐to‐roll made PET@UiO‐66‐NH_2_, PET, and carbon cloth is shown in Figure 4b. The weight of PET and PET@UiO‐66‐NH_2_ was increased by stacking 5 cm ×5 cm swatches to form 1, 2, or 3 layers of fabrics. One layer of PET, PET@UiO‐66‐NH_2_, and carbon cloth weigh approximately 79, 110, and 172 gsm, respectively. Breakthrough was instantaneous with 3 layers of untreated PET. PET@UiO‐66‐NH_2_ significantly outperformed the untreated PET, and the breakthrough time increased with increasing layers (gsm) of PET@UiO‐66‐NH_2_. Three layers of PET@UiO‐66‐NH_2_ performed better as a barrier to GD than carbon cloth. PET@UiO‐66‐NH_2_ performed similarly or better than previous reports of solid‐state hydrolysis of soman using UiO‐66‐NH_2_ fabrics when comparing time to full GD conversion.[^9^, ^34^, ^38^, ^39^] A more detailed comparison of effects of different MOF loadings, crystal size, fabric properties, and others would be helpful to further understand the detailed mechanisms and criteria for GD degradation upon exposure to MOF‐fabrics. With both degradation and barrier capabilities, our MOF‐fabrics outperform the long‐standing standard for organophosphate protection.

## Conclusion

In this report, we explored UiO‐66‐NH_2_ synthesis in benign solvents and presented a mechanism for linker dissolution and rapid metal–organic framework (MOF) formation. By separating the linker and metal in co‐compatible solutions, we avoided premature side reactions and promoted heterogeneous MOF nucleation in both batch and roll‐to‐roll synthesis of UiO‐66‐NH_2_‐fabric composites. Our benign solvent choice allowed rapid post processing (“fast‐wash”) of the MOF‐fabric without sacrificing material properties compared to more conventional methods (“slow‐wash”). PET@UiO‐66‐NH_2_ composites showed full conversion of soman in 24 h under solid‐state conditions and performed as an effective barrier for soman permeation. The approaches and chemical insights provided by this work expand Zr‐MOF‐fabric synthesis approaches to industrially relevant techniques and have wide impact due to the multitude of applications currently being explored for MOF‐fabrics. This simple, scalable MOF‐fabric synthesis method provides a significant step towards the goal of next generation, feature enhanced, affordable personal protective equipment.

## Experimental Section

### Materials

Solvents included deionized water (H_2_O, in‐house), ethanol (EtOH, 200 proof Koptec), and glacial acetic acid (HOAc, >99.7 % Northwest Scientific Inc.). Zirconium(IV)chloride (ZrCl_4_, 99.5 % Alfa Aesar) and 2‐aminoterephthalic acid (BDC‐NH_2_, 99 % Aldrich) were used as UiO‐66‐NH_2_ precursors. All solvents and precursors were used without further purification. Fabrics used include nonwoven polyethylene terephthalate (PET), polypropylene (PP) and nylon (PA‐6) (The Nonwovens Institute), 50 : 50 nylon/cotton (nyco) (U.S. Army Natick Soldier Systems Center), and spandex(8 %)/polyester (spandex) (commercial garment). All fabrics were used as received without further treatment.

### UiO‐66‐NH_2_ powder synthesis

0.13 m BDC‐NH_2_ was dispersed in equal parts H_2_O/HOAc via sonication in a media vial and heated to 90 °C. In a separate vial, 0.14 m ZrCl_4_ was dissolved in EtOH by stirring at room temperature. While stirring separately, 2 mL of the metal solution followed by 2 mL heated linker solution were added to a new vial, stirred for around 30 s, and the vial was placed in a preheated oven at 90 °C. The vial was removed from the oven at a designated time point, the product was stirred for around 5 s, then 100 μL of the product solution was placed on an IR transparent silicon (Si) wafer and dried at room temperature. After visual inspection, the remaining product solution was quenched with room temperature EtOH. The product was then separated by filtration, washed in fresh EtOH 10 min, re‐filtered, dried 30 min at 120 °C, and, finally, dried 60 min at 85 °C under vacuum.

### SS SVS of fabric@UiO‐66‐NH_2_

First, approximately 2.6 mL of 0.13 m BDC‐NH_2_ solution and 2.6 mL of 0.14 m ZrCl_4_ solution, previously described in powder experimental section, were sprayed simultaneously onto a 2 in ×2 in fabric swatch. Next, the fabric was suspended in a media vial containing room temperature water, and the vial was immediately transferred to a 90 or 120 °C preheated oven. After heating for 0.5–3 h, the vial was removed from the oven and cooled for 10 min at room temperature. The fabric was then removed from the vial, washed in EtOH, and dried using either a slow or rapid method. The more conventional, slow method consisted of a short 5 min soak in EtOH immediately followed by a slow 18–24 h soak in fresh EtOH, followed by drying in air at 75 °C for 18–24 h and drying under vacuum at 85 °C for 18–24 h. The resulting samples are referred to here as “slow‐made” MOF‐fabric. The rapid method consisted of soaking the MOF‐fabric in EtOH for 5 min, transferring to a second soak in fresh EtOH for another 5 min, then drying in air at 75 °C for 30 min, followed by drying under vacuum at 85 °C for 1 h. These samples are referenced as “rapid‐made” MOF‐fabric.

### Roll‐to‐roll PET@UiO‐66‐NH_2_ synthesis

Roll‐to‐roll PET@UiO‐66‐NH_2_ production was conducted on a home‐built roll‐to‐roll line. A long, 6” (15 cm) wide PET fabric piece was suspended between two rollers with excess PET on the starting roll. First, 40 mL 0.13 m BDC‐NH_2_ in equal parts H_2_O/HOAc was sonicated for around 20 min or until use. 40 mL 0.14 m ZrCl_4_ in EtOH was prepared and stirred until use. The linker and metal solutions were combined into a single spray bottle and immediately sprayed onto around 8” (20 cm) of the PET roll. The sprayed section of fabric was then immediately rolled into a heat/vapor zone (90–110 °C) where it remained for 1 h. When the 1 h was complete, the next section of fabric was sprayed with precursor solution and rolled into the heat/vapor zone as the previous section was rolled from the heat/vapor zone onto the final MOF‐fabric roll. After several iterations, the MOF‐fabric was cut from the starting roll, washed in EtOH for 5 min, removed and washed in fresh EtOH an additional 5 min, dried at 75 °C 18–24 h, and dried at 85 °C under vacuum 1 h.

### MOF‐fabric robustness tests

Tape tests were performed by placing scotch tape on the MOF‐fabric. The tape was then quickly removed and visually inspected for MOF powder contaminates. We also performed an abrasion test which consisted of vigorously shaking the MOF‐fabric in a 100 mL media vial with 120 g of metal balls for 1 min. The fabric was weighed before and after the abrasion test to determine mass loss.

### Methyl paraoxon hydrolysis

Methyl paraoxon (DMNP) hydrolysis tests were performed as previously described.^[9]^ 14 mg untreated fabric or MOF‐fabric, 1 mL 0.45 m aqueous *N*‐ethylmorpholine (NEM, Fluka), and a stir bar were placed in a Eppendorf tube, weighed, then stirred at around 1100 rpm for 15 min. 4 μL DMNP (Pestanal) was then added to the Eppendorf tube wall without touching the NEM solution or MOF‐fabric, and the tube was weighed to obtain the weight of DMNP. The tube was then shaken for several seconds to incorporate the DMNP into the solution. The mixture was then continuously stirred. At designated time points, 20 μL aliquots of the reaction solution were diluted in 10 mL 0.15 m aqueous NEM solution. UV/Vis spectroscopy (Thermo Scientific 300) was used to track formation of the product *p*‐nitrophenoxide at around 407 nm. DMNP half‐life (*t*_1/2_) and turnover frequency at half‐life were (TOF_*t*1/2_) were calculated as previously detailed.^[9]^

### Soman solid‐state hydrolysis

MOF‐fabrics were tested using dose extraction techniques to the solid substrate. Untreated fabric or MOF‐fabric (10–40 mg) was first placed in a small vial to minimize vapor loss. Vials were then placed in a preheated oven and dried at 60 °C for 1 h followed by exposure to 50 % relative humidity overnight. Soman was then pipetted directly onto the fiber surface and the vial vortexed to ensure maximum contact. After a set time, vials were cooled with dry ice for 10 min and 1–2 mL acetonitrile was used to extract reactants and products from the fabric. Extraction solvent was passed through a 0.45 μm nylon membrane syringe filter, and the amount of soman remaining was determined by GC/MS (Agilent 6890/5973). Warning! Soman is an extremely toxic chemical warfare agent and should only be handled by authorized personnel.

### Soman permeation

Soman permeation was conducted in accordance with ASTM F739‐12 as previously reported.^[9]^ Fabric was first loaded into a 1 in diameter Pesce PTC 700 permeation test cell. Two air streams, one above the sample and one below, were maintained at a flow rate of 300 mL min^−1^ and 0 % relative humidity. A constant 0.2 in of H_2_O pressure differential was maintained across the samples. The feed stream contained 300 mg m^−1^ of GD, and GD concentration was monitored at three locations within the permeation cell including the inlet, outlet, and opposite side of the permeation cell. Concentration was measured by GC with a flame ionization detector. Warning! Soman is an extremely toxic chemical warfare agent and should only be handled by authorized personnel.

### Characterization

MOF mass loading (mass of MOF divided by mass of composite) was determined by weighing the fabrics before synthesis and after washing and drying was complete. SEM images were collected using an FEI Verios 460 L field‐emission scanning electron microscope. Samples were sputter coated with around 14 nm Au/Pd prior to imaging. XRD patterns were collected using a Rigaku SmartLab X‐ray diffractometer under Bragg‐Brentano alignment and a Cu K_α_ source. A Micrometrics 3Flex Physisorption was used to collect N_2_ isotherm data. Samples were prepared for N_2_ isotherm analysis by holding at 100 °C under vacuum overnight using a Micrometrics Smart Vac Prep system. FTIR spectra were collected using a Nicolet 6700 FTIR. IR Si was used as the substrate and background for FTIR measurements. XPS data was collected using Kratoc Analytical Axis Ultra with an Al K_α_ gun operating at 15 kV and 10 mA.

## Conflict of interest

The authors declare no conflict of interest.

1

## Supporting information

As a service to our authors and readers, this journal provides supporting information supplied by the authors. Such materials are peer reviewed and may be re‐organized for online delivery, but are not copy‐edited or typeset. Technical support issues arising from supporting information (other than missing files) should be addressed to the authors.

Supporting Information

Supporting Information

## Data Availability

The data that support the findings of this study are available from the corresponding author upon reasonable request.
