# Supplementary material for: Toxin‐Blocking Textiles: Rapid, Benign, Roll‐to‐Roll Production of Robust MOF‐Fabric Composites for Organophosphate Separation and Hydrolysis
Source: ChemSusChem. 2022 Nov 18;16(2):e202201744. doi: 10.1002/cssc.202201744 (PMC10100493; doi:10.1002/cssc.202201744)
Supplement: Supplementary file 1 — Supporting Information [file CSSC-16-0-s002.pdf]

ChemSusChem

Supporting Information

Toxin-Blocking Textiles: Rapid, Benign, Roll-to-Roll Production of Robust MOF-Fabric Composites for Organophosphate Separation and Hydrolysis

Sarah E. Morgan, Morgan L. Willis, Golnaz Dianat, Gregory W. Peterson, John J. Mahle, and Gregory N. Parsons*© 2022 The Authors. ChemSusChem published by Wiley-VCH GmbH. This is an open access article under the terms of the Creative Commons Attribution License, which permits use, distribution and reproduction in any medium, provided the original work is properly cited.

Table of Contents

Table S1 2
Table S2 3
Figure S1 3
Figure S2 4
Figure S3 5
Figure S4 6
Figure S5 7
Figure S6 7
Figure S7 8
References 9

Table S1. Summary of green synthesis methods for UiO-66 type powder in literature.

MOF	Primary solvents/ modulators	T [°C]	Reaction Conditions	Time [h]				Ref.
				Synthesis	Washing	Drying	**Total**	
UiO-66-NH_2	H_2O/EtOH/ HOAc	90	-	0.24 - 1	0.2	1.5	**3**	This work
UiO-66-$(OH)_2$ ǂ	H_2O/HOAc	95	Stirring/ microwave	1	N/A	24	**25**	[1]
UiO-66-NO_2	H_2O/HOAc	RT	stirring	72	48	48	**168**	[2]
UiO-66	IL/HOac	RT	stirring	1	4	N/A	**5**	[3]
UiO-66	IL/HOAc/ H_2O	120	ionothermal	24	5	N/A	**29**	[3]
UiO-66$(COOH)_2$	H_2O/HOAc	130	reflux	10	N/A	12	**22**	[4]
UiO-66-NH_2 ǂ	H_2O/HOAc	RT	stirring	72	N/A	12	**84**	[5]
UiO-66-(COOH) ǂ	H_2O/HOAc	90	stirring	24	N/A	N/A	**24**	[5]
UiO-66-NH_2	H_2O/HOAc	90-150	Continuous flow	-	24	12	**36**	[6]
UiO-66-NH_2	H_2O/HOAc	90	Stirring	24	72	24	**120**	[7]
UiO-66	Solvent free	130	Autoclave	12	N/A	12	**24**	[8]
UiO-66-PYDC	H_2O/HOAc	100	-	24	N/A	N/A	**24**	[9]
UiO-66-F_4 ǂ	H_2O	-	Liquid assisted grinding	0.25	N/A	24	**24**	[10]
UiO-66 ǂ	H_2O/formic acid	98	stirring	16	N/A	N/A	**16**	[11]
hcp UiO-66	H_2O/Benz or formic acud/	100	-	72	N/A	24	**96**	[12]
UiO-66-NH_2–F_4	H_2O /HOAc	100	reflux	24	144	24	**192**	[13]
UiO-66 ǂ	H2O/HOAc or formic or TFA	105	reflux	24	96	24	**144**	[14]
UiO-66 ǂ	H_2O/HOAc	100/120	reflux	24	144	24	**192**	[15]
UiO-66-F_4	H_2O/HOAc	120/150	autoclave	24	N/A	N/A	**24**	[16]
UiO-66	IL/HOAc	RT	stirring	0.17 - 1	48	24	**73**	[17]
UiO-66-$(COOH)_2$ ǂ	H_2O/TFA or HOAc	RT	stirring	40-48	48	N/A	**96**	[18]
hcp UiO-66	H_2O/ HOAc/IL	100	-	72	N/A	24	**96**	[19]
UiO-66-$(COOH)_2$	H_2O	100	reflux	24	16	N/A	**40**	[20]
UiO-66	H_2O/NaOH	RT	Dropping/ stirring	24	N/A	N/A	**24**	[21]
UiO-66 ǂ	H_2O/TEA	-	Milling	0.5-1.5	N/A	N/A	**1.5**	[22]
UiO-66-NH_2	NaOH/H_2O/ HOAc	RT	stirring	1-24	16	16	**33**	[23]
UiO-66-(COOH) ǂ	H_2O	110-150	reflux	24	16	N/A	**40**	[24]

RT: room temperature; N/A: time not given; ǂ other UiO-66 analogs were also synthesized

Table S2. Summary of *in-situ* fiber-first UiO-66-NH_2 fabrics in literature.

Substrate	Benign	Primary solvent	Time [h]					Ref.
			Pretreatment	Synthesis	Washing	Drying	**Total**	
PET	✓	H_2O/EtOH/HOAc	0	1	0.2	1.5	**2.7**	This work
PP	X	DMF	2.5	24	48	12	**86.5**	[25]
Spandex	X	DMF	0	3	48	48	**99**	[26]
Spandex	✓	GVL	0	24	24	48	**96**	[27]
PET	X	DMF	15	24	N/A	N/A	**39**	[28]
Cotton	✓	H_2O/NaOH	N/A	< 1h	< 1h	24	**26**	[29]
Graphene oxide	X	DMF	0	24	N/A	12	**26**	[30]
PET	✓	H_2O/TFA	0	4	>24	24	**52**	[31]
Cotton	X	DMF	2	20	N/A	12	**34**	[32]
Cotton	X	DMF	12	24-120	N/A	24	**60**	[33]
PAN	✓	Acetone	0-24	24	1.5	0.5	**26**	[34]
PP	X	DMF	8	24	48	24	**104**	[35]
PMMA	X	DMF	5	24	50	12	**91**	[36]
Cotton	X	DMF	96	24	N/A	N/A	**120**	[37]
Cotton	X	DMF	4	24	0.5	24	**52.5**	[38]
Cotton/Polyamide	X	DMF	0	1.2	N/A	N/A	**1.2**	[39]

N/A: time not given

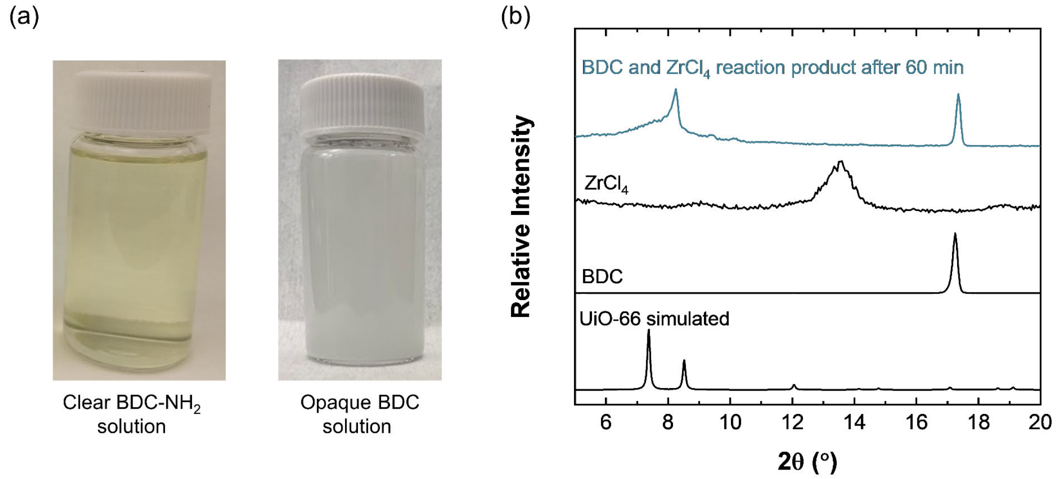

Figure S1. (a) Optical images of BDC-NH_2 and BDC dissolved in heated H_2O/HOAc/EtOH/HCl solution; (b) XRD of BDC and $ZrCl_4$ powder and dried reaction product of BDC and $ZrCl_4$.

(a)

Vapor exposure time [h]	Vapor exposure temp [°C]	MOF mass loading [%]	BET SA [$m^2\ g_{comp}^{-1}$]	Porous MOF loading [%]	DMNP $t_{1/2}$ [min]	$TOF_{t½}$ [min^{-1}]
0.5	90	16	109	10	-	-
1	90	16	119	11	35	0.1
3	90	17	113	10	19	0.2
0.5	120	19	98	8	40	0.4
1	120	16 ± 1	123 ± 14	15 ± 2	18 ± 4	0.34 ± 0.05

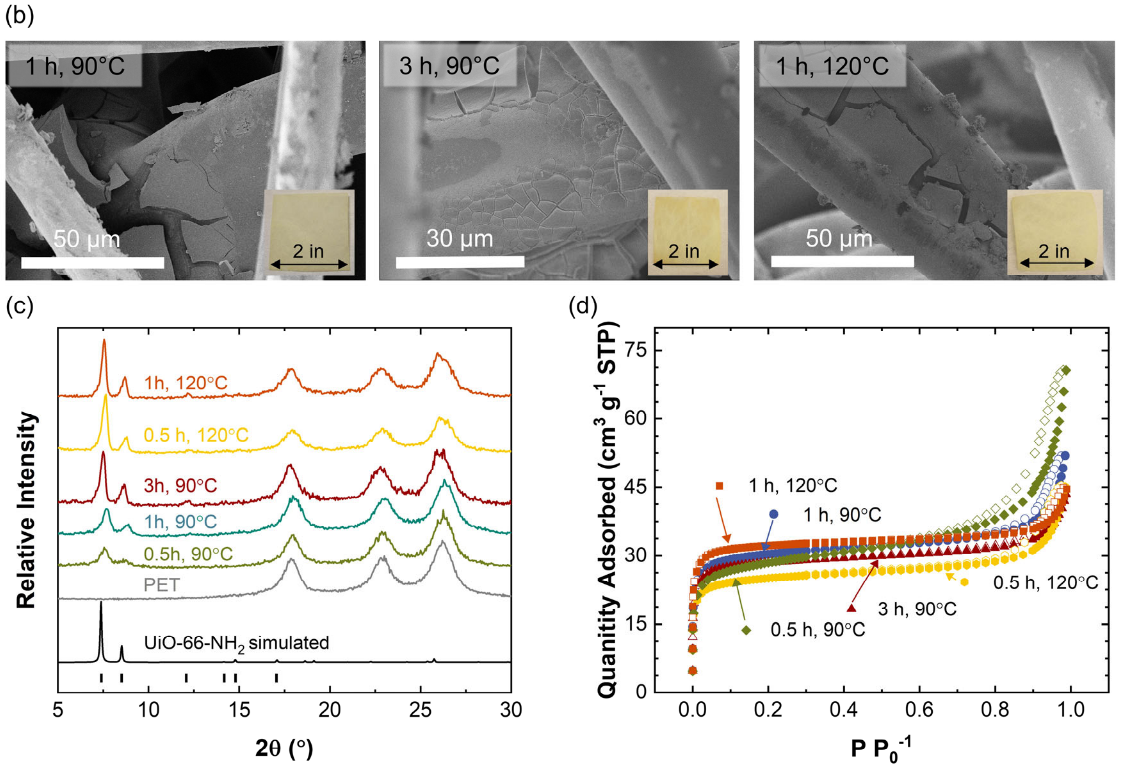

Figure S2. (a) Table of select properties, (b) SEM images, (c) XRD patterns, and (d) N_2 isotherms of slow-made PET@UiO-66-NH_2 made with various vapor exposure times and temperatures.

Slow-made PET@UiO-66-NH_2 was synthesized by SS-SVS at 90°C with a vapor exposure time (Step 2) of 0.5, 1, or 3 h, and the resulting composites were analyzed and compared as shown in Figure S2. MOF loading was between 16-17 wt% for all vapor exposure times studied. As shown in Figure S2a, compared to the white starting PET fabric, all MOF-fabric samples appear yellow consistent with UiO-66-NH_2 on the fabric surface. XRD patterns indicate that the relative crystallinity of the MOF coating increases with vapor exposure time. N_2 isotherms were comparable for all vapor exposure times studied and resulted in BET SA and porous MOF loading between 109 – 120 $m^2\ g_{comp}^{-1}$ and 10-11 wt%, respectively. DMNP $t_{1/2}$ significantly improved with increase in vapor exposure time. Uncoated PET had minimal reactivity towards DMNP in 90 min. Based on the data in Figure S2, we hypothesize that the reactants are fully consumed within the first 30 minutes of the heating process to create a crystalline product. Continued heating leads to more extensive crystallization, producing larger crystal diffractions observed by XRD. Interestingly, the less crystallized materials formed after the first 30 min show porosity and surface area similar to more crystalline material formed after

3 h of heating. This suggests that after 30 min, the MOF material consists of either ultra-small crystals that are less visible by XRD, or the MOF forms initially in a porous, amorphous structure. Results also indicate that an increase in crystallinity leads to improved DMNP hydrolysis kinetics.

Vapor exposure temperature was set at 90 or 120°C with an exposure time of 1 h for SS SVS of PET@UiO-66-NH_2, and composites are compared in Figure S2. MOF-fabrics were visually similar, and MOF loading was consistent for the temperatures studied. UiO-66-NH_2 XRD peak intensity significantly increased with vapor exposure temperature. Resulting N_2 isotherms composite BET SAs, and porous MOF loadings were consistent between the two temperatures studied. DMNP $t_{1/2}$ was significantly enhanced from 35 to 13 min with increase in reaction temperature from 90 to 120°C, respectively. Based on the temperature dependent results, we hypothesize that the crystallization speed is proportional to vapor exposure temperature. Increased vapor exposure temperature leads to a product with larger crystal diffractions as observed by XRD. Observations also further support the hypothesis that DMNP hydrolysis rates increase with increase in MOF coating crystallinity.

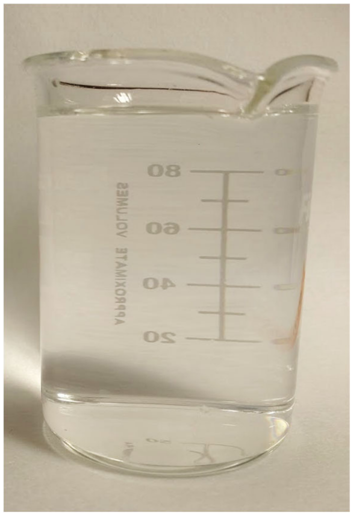

Figure S3. Optical image of EtOH after being used to wash PET@UiO-66-NH_2.

(a)

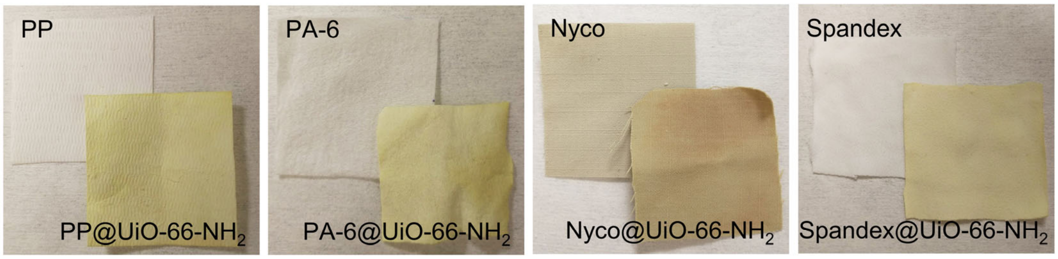

(b)

Fabric	MOF loading [wt%]	BET SA [$m^2\ g_{comp}^{-1}$]	MOF BET loading [wt%]
PP	15	22	2
PA-6	10	55	6
Nyco	1	12	1
Spandex	5	43	5
PET	17	130	12

Figure S4. (a) Images and (b) select properties of rapid-made fabric@UiO-66-NH_2.

Rapid SS-SVS with 1 h and 120°C vapor exposure time and temperautre was used to form UiO-66-NH_2 coated nonwoven polypropylene (PP) and nylon (PA-6) as well as woven 50/50 nylon/coton (nyco) and 8/92 spandex/polyester (spandex) fabrics. All fabrics had significant color change after MOF growth shown in Figure S4a. The data shows that MOF loading and BET SA depend heavily on fabric type. The PP had the largest loading at 15 wt%, and nyco had the smallest at 1 wt%. Interestingly, PA-6 had 10x higher loading than nyco even though nyco is composed of 50% nylon. The MOF loading calculated by mass was larger than the porous MOF loading for each fabric and greatly depended on fabric type.

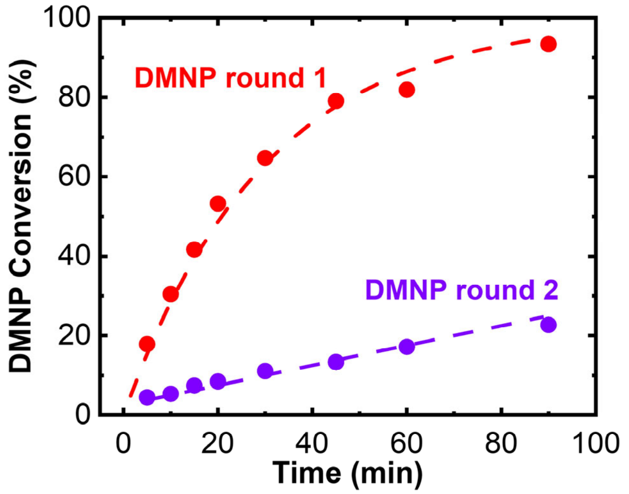

Figure S5. Round 1 and round 2 DMNP conversion with rapid-made PET@UiO-66-NH_2

Figure S5 shows the results from two rounds of DMNP hydrolysis completed using the same rapid-made PET@UiO-66-NH_2 sample. DMNP hydrolysis was run in the same manner as listed in the Experimental using 14 mg MOF-fabric. After reacting with the DMNP for 90 min (round 1), the fabric was removed from the solution, washed in H_2O for 12 min (replacing the H_2O 3 times), washed in EtOH for 10 min (replacing 2 EtOH time), and dried for 24 h at 75°C followed by 30 min at 85°C under vacuum to re-activate the MOF. The same piece of MOF-fabric was then used for other DMNP hydrolysis experiment (round 2). We find that the half-life significantly increased from 18 to 225 min. The turn-over frequency at 20% DMNP conversion for the first and second DMNP experiment were 0.4 and 0.2 min^{-1}, respectively. The MOF fabric can be reset and reused. However, there is decrease in performance that is likely attributed to MOF loss during the experiment due to vigorous, maintained stirring.

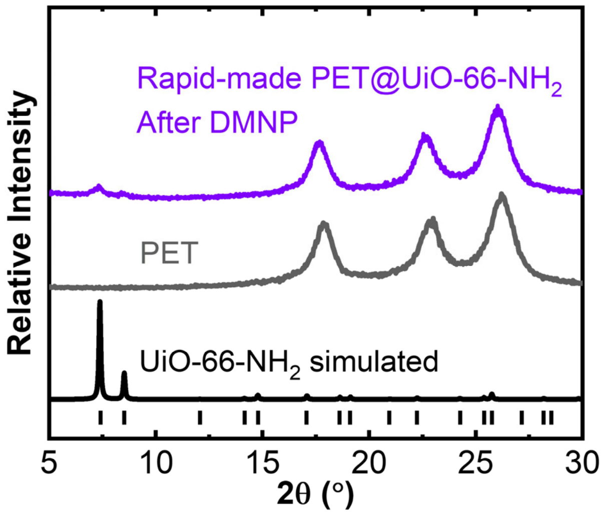

Figure S6. XRD of rapid-made PET@UiO-66-NH2 after DMNP hydrolysis.

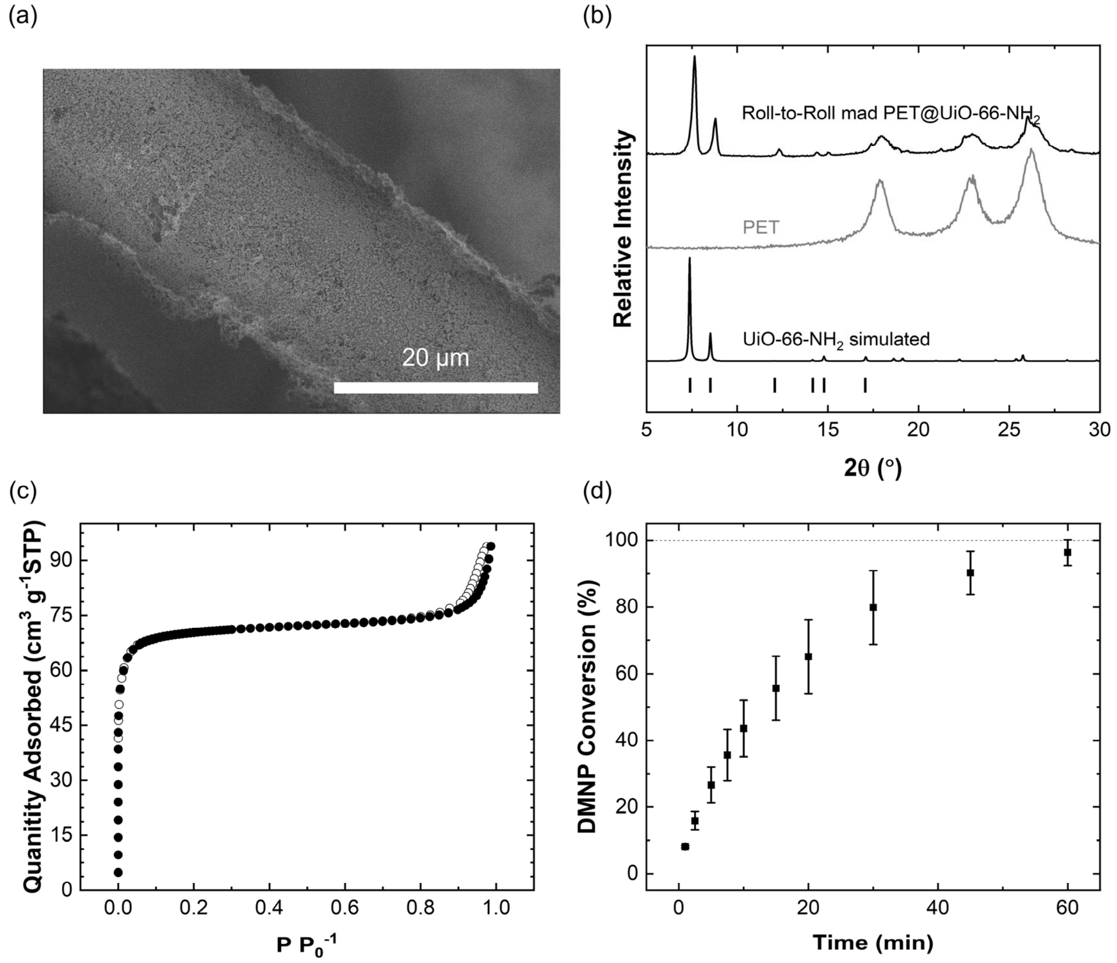

Figure S7. (a) SEM image, (b) XRD pattern, (c) N_2 isotherm, and (d) DMNP conversion plot of roll-to-roll made PET@UiO-66-NH_2.

References

[1] H. Reinsch, S. Waitschat, S. M. Chavan, K. P. Lillerud, N. Stock, *Eur. J. Inorg. Chem.* **2016**, *2016*, 4490–4498.

[2] Z. Chen, X. Wang, T. Islamoglu, O. K. Farha, *Inorganics* **2019**, *7*, 2–3.

[3] F. Joly, P. Devaux, T. Loiseau, M. Arab, B. Morel, C. Volkringer, *Microporous Mesoporous Mater.* **2019**, *288*, 109564–109570.

[4] M. N. Nimbalkar, B. R. Bhat, *Mater. Today Proc.* **2019**, *9*, 522–527.

[5] C. Avci-camur, J. Pérez-Carvajal, I. Imaz, D. Maspoch, *ACS Sustain. Chem. Eng.* **2018**, *6*, 14554–14560.

[6] C. Avci-camur, J. Troyano, J. Pérez-Carvajal, A. Legrand, D. Farrusseng, I. Imaz, D. Maspoch, *Green Chem.* **2018**, *20*, 873–878.

[7] Y. Zhang, B. Li, Y. Wei, C. Yan, M. Meng, Y. Yan, *J. Taiwan Inst. Chem. Eng.* **2019**, *96*, 93–103.

[8] G. Ye, D. Zhang, X. Li, K. Leng, W. Zhang, J. Ma, Y. Sun, W. Xu, S. Ma, *ACS Appl. Mater. Interfaces* **2017**, *9*, 34937–34943.

[9] Z. Wang, Y. Huang, J. Yang, Y. Li, Q. Zhuang, J. Gu, *Dalt. Trans.* **2017**, *46*, 7412–7420.

[10] Y. Huang, W. Lo, Y. Kuo, W. Chen, C. Lin, F. Shieh, *ChemComm* **2017**, *53*, 5818–5821.

[11] H. Reinsch, B. Bueken, F. Vermoortele, I. Stassen, A. Lieb, K. Lillerud, D. De Vos, *CrystEngComm* **2015**, *17*, 4070–4074.

[12] M. Ermer, J. Mehler, M. Kriesten, Y. S. Avadhut, P. S. Schulz, M. Hartmann, *Dalt. Trans.* **2018**, *47*, 14426–14430.

[13] Z. Hu, A. Gami, Y. Wang, D. Zhao, *Adv. Sustain. Syst.* **2017**, *1*, 1700092–1700104.

[14] Z. Hu, I. Castano, S. Wang, Y. Wang, Y. Peng, Y. Qian, C. Chi, X. Wang, D. Zhao, *Cryst. Growth Des.* **2016**, *16*, 2295–2301.

[15] Z. Hu, Y. Peng, Z. Kang, Y. Qian, D. Zhao, *Inorg. Chem.* **2015**, *54*, 4862–4868.

[16] F. C. N. Firth, M. J. Cliffe, D. Vulpe, M. Aragones-Anglada, P. Z. Moghadam, D. Fairen-Jimenez, B. Slater, C. P. Grey, *J. Mater. Chem. A* **2019**, *7*, 7459–7469.

[17] X. Sang, J. Zhang, J. Xiang, J. Cui, L. Zheng, J. Zhang, Z. Wu, Z. Li, G. Mo, Y. Xu, J. Song, C. Liu, X. Tan, T. Luo, B. Zhang, B. Han, *Nat. Commun.* **2017**, *8*, 175.

[18] Z. Chen, X. Wang, H. Noh, G. Ayoub, G. W. Peterson, C. T. Buru, T. Islamoglu, O. K. Farha, *CrystEngComm* **2019**, *21*, 2409–2415.

[19] M. Ermer, J. Mehler, B. Rosenberger, M. Fischer, P. S. Schulz, M. Hartmann, *ChemistryOpen* **2021**, *10*, 233–242.

[20] Q. Yang, S. Vaesen, F. Ragon, A. D. Wiersum, D. Wu, A. Lago, T. Devic, C. Martineau, F. Taulelle, P. L. Llewellyn, H. Jobic, C. Zhong, C. Serre, G. De Weireld, G. Maurin, *Angew. Chemie - Int. Ed.* **2013**, *52*, 10316–10320.

[21] J. M. Yassin, A. M. Taddesse, M. Sánchez-Sánchez, *Catal. Today* **2022**, *390–391*, 162–175.

[22] B. Karadeniz, A. J. Howarth, T. Stolar, T. Islamoglu, I. Dejanović, M. Tireli, M. C. Wasson, S. Y. Moon, O. K. Farha, T. Friščić, K. Užarević, *ACS Sustain. Chem. Eng.* **2018**, *6*, 15841–15849.

[23] I. Pakamore, J. Rousseau, C. Rousseau, E. Monflier, P. Á. Szilágyi, *Green Chem.* **2018**, *20*, 5292–5298.

[24] F. Ragon, B. Campo, Q. Yang, C. Martineau, A. D. Wiersum, A. Lago, V. Guillerm, C. Hemsley, J. F. Eubank, M. Vishnuvarthan, F. Taulelle, P. Horcajada, A. Vimont, P. L. Llewellyn, M. Daturi, S. Devautour-Vinot, G. Maurin, C. Serre, T. Devic, G. Clet, *J. Mater. Chem. A* **2015**, *3*, 3294–3309.

[25] D. T. Lee, Z. Dai, G. W. Peterson, M. G. Hall, N. L. Pomerantz, N. Hoffman, G. N. Parsons, *Adv. Funct. Mater.* **2022**, *32*, 2108004–2108016.

[26] S. E. Morgan, A. M. O'Connell, A. Jansson, G. W. Peterson, J. J. Mahle, T. B. Eldred, W. Gao, G. N. Parsons, *ACS Appl. Mater. Interfaces* **2021**, *13*, 31279–31284.

[27] S. E. Morgan, M. L. Willis, G. W. Peterson, J. J. Mahle, G. N. Parsons, *ACS Sustain. Chem. Eng.* **2022**, *10*, 2699–2707.
[28] F. Zhao, C. Su, W. Yang, Y. Han, X. Luo, C. Li, W. Tang, T. Yue, Z. Li, *Appl. Surf. Sci.* **2020**, *527*, 146862–146872.
[29] L. Huelsenbeck, H. Luo, P. Verma, J. Dane, R. Ho, E. Beyer, H. Hall, G. M. Geise, G. Giri, *Cryst. Growth Des.* **2020**, *20*, 6787–6795.
[30] L. Song, T. Zhao, D. Yang, X. Wang, X. Hao, Y. Liu, S. Zhang, Z.-Z. Yu, *J. Hazard. Mater.* **2020**, *393*, 122332–122344.
[31] K. Ma, T. Islamoglu, Z. Chen, P. Li, M. C. Wasson, Y. Chen, Y. Wang, G. W. Peterson, J. H. Xin, O. K. Farha, *J. Am. Chem. Soc.* **2019**, *141*, 15626–15633.
[32] D. K. Yoo, S. H. Jhung, *ACS Appl. Mater. Interfaces* **2019**, *11*, 47649–47657.
[33] J. Hyunsook, M.-K. Kim, S. Jang, *J. Colloid Interface Sci.* **2020**, *563*, 363–369.
[34] A. X. Lu, A. M. Ploskonka, T. M. Tovar, G. W. Peterson, J. B. Decoste, *Ind. Eng. Chem. Res.* **2017**, *56*, 14502–14506.
[35] N. L. Pomerantz, E. E. Anderson, N. P. Dugan, N. F. Hoffman, H. F. Barton, D. T. Lee, C. J. Oldham, G. W. Peterson, G. N. Parsons, *ACS Appl. Mater. Interfaces* **2019**, *11*, 24683–24690.
[36] D. B. Dwyer, D. T. Lee, S. Boyer, W. E. Bernier, G. N. Parsons, W. E. Jones, *ACS Appl. Mater. Interfaces* **2018**, *10*, 25794–25803.
[37] M. A. Bunge, A. B. Davis, K. N. West, C. W. West, T. G. Glover, *Ind. Eng. Chem. Res.* **2018**, *57*, 9151–9161.
[38] M.-K. K. Kim, S. H. Kim, M. Park, S. G. Ryu, H. Jung, *RSC Adv.* **2018**, *8*, 41633–41638.
[39] N. Couzon, M. Ferreira, S. Duval, A. El-Achari, C. Campagne, T. Loiseau, C. Volkringer, *ACS Appl. Mater. Interfaces* **2022**, *14*, 21497−21508.
